# GeneAnalytics Pathway Analysis and Genetic Overlap among Autism Spectrum Disorder, Bipolar Disorder and Schizophrenia

**DOI:** 10.3390/ijms18030527

**Published:** 2017-02-28

**Authors:** Naveen S. Khanzada, Merlin G. Butler, Ann M. Manzardo

**Affiliations:** 1 Department of Psychiatry and Behavioral Sciences, University of Kansas Medical Center, Kansas City, KS 66160, USA; nkhanzada@kumc.edu (N.S.K.); mbutler4@kumc.edu (M.G.B.); 2 Department of Pediatrics, University of Kansas Medical Center, Kansas City, KS 66160, USA

**Keywords:** mental illness, genetic profiling, GeneAnalytics molecular pathway analysis, circadian entrainment

## Abstract

Bipolar disorder (BPD) and schizophrenia (SCH) show similar neuropsychiatric behavioral disturbances, including impaired social interaction and communication, seen in autism spectrum disorder (ASD) with multiple overlapping genetic and environmental influences implicated in risk and course of illness. GeneAnalytics software was used for pathway analysis and genetic profiling to characterize common susceptibility genes obtained from published lists for ASD (792 genes), BPD (290 genes) and SCH (560 genes). Rank scores were derived from the number and nature of overlapping genes, gene-disease association, tissue specificity and gene functions subdivided into categories (e.g., diseases, tissues or functional pathways). Twenty-three genes were common to all three disorders and mapped to nine biological Superpathways including Circadian entrainment (10 genes, score = 37.0), Amphetamine addiction (five genes, score = 24.2), and Sudden infant death syndrome (six genes, score = 24.1). Brain tissues included the medulla oblongata (11 genes, score = 2.1), thalamus (10 genes, score = 2.0) and hypothalamus (nine genes, score = 2.0) with six common genes (*BDNF*, *DRD2*, *CHRNA7*, *HTR2A*, *SLC6A3*, and *TPH2*). Overlapping genes impacted dopamine and serotonin homeostasis and signal transduction pathways, impacting mood, behavior and physical activity level. Converging effects on pathways governing circadian rhythms support a core etiological relationship between neuropsychiatric illnesses and sleep disruption with hypoxia and central brain stem dysfunction.

## 1. Introduction

Severe neuropsychiatric disorders collectively present with similar behavioral, social, cognitive and perceptual disturbances including autism spectrum disorder (ASD). ASD includes classical autism, Asperger syndrome and pervasive developmental disorder with problems in social interaction and communication or repetitive behavior. Schizophrenia (SCH) presents with delusions, hallucinations, disorganized thinking and behavior with negative symptoms. Bipolar disorder (BPD) is considered a developmental disorder characterized by progressive cognitive impairment, residual symptoms, sleep disturbance, and emotional dysregulations with cycles of depression and mania. Schizophrenia and bipolar disorders share many common traits with ASD, including social and cognitive dysfunction and impaired ability to function, live and work independently [1]. Considerable overlap has been identified between the molecular mechanisms implicated in the etiology of schizophrenia, bipolar disorder, and autism, suggesting similar root causes [2]. Up to 30% of patients diagnosed with ASD during childhood will develop schizophrenia during adulthood [3]. Further, the presence of schizophrenia or bipolar disorder in first-degree relatives is a consistent and significant risk factor for ASD [4]. These three neuropsychiatric illnesses have complex inheritance patterns with >80% estimate for each disorder with multiple genetic and environmental factors influencing disease risk and course [2,4].

A recent large collaborative genetic study of families with schizophrenia and ASD showed significant overlap in candidate genes and susceptibility regions for both disorders using traditional karyotyping, genome-wide association studies (GWAS) and comparative genome hybridization (CGH) analyses by identifying chromosomal deletions and duplications in individuals with ASD [5,6,7,8]. ASD and SCH risk alleles appear to impact growth-signaling pathways with autism associated with loss of function in many genes [9,10,11], whereas schizophrenia tends to be associated with reduced function or activity of genes that up-regulate growth-related pathways [12,13,14,15,16].

Cytogenetic, linkage and association studies have identified common copy number variants (CNVs, deletions, duplications) between ASD and SCH which may produce dosage-dependent gain or loss of expression of genes contributing to phenotypic variation in presentation and course of illness. A large number of autism-specific CNVs have been found but with low recurrence (<1%) and they show a high level of genetic heterogeneity. For example, when the 15q11.2 BP1–BP2 region or 15q13.3 band contains a deletion or duplication in patients, then autism or a variety of neuropsychiatric traits including schizophrenia are identified [3,17,18,19]. Gene expression disturbances were found using postmortem cortical brain tissue from patients with autism, schizophrenia and bipolar disorder and they have shown a high correlation between the transcriptomes of ASD and schizophrenia, but not in BPD [5]. Hence, a large number of possible genetic and environmental factors do influence disease risk, expression and treatment.

Herein, we use the GeneAnalytics [20] program pathway analysis to further profile and characterize the underlying molecular architecture of clinically and etiologically relevant genes common to ASD [21], bipolar disorder [22] and schizophrenia [23] and associated diseases.

## 2. Results

The original gene lists reported in the literature included 792 genes for ASD [21], 290 genes for bipolar disorder [22] and 560 genes for schizophrenia [23], and of these, 23 genes were found in common in all three conditions (see Table 1). Functional analysis of the 23 genes identified from the submitted list of genes showed a high match for schizophrenia (17 genes, score = 15.1) with medium-match scores representing 25 other disorders including bipolar disorder (nine genes, score = 9.6) and autism spectrum disorder (10 genes, score = 9.1). Additional diseases identified were related to disorders of mental health including mood and personality disorders (see Table 2A). Tissues and cell types profiled for these 23 overlapping genes identified five common types of brain tissues which achieved high match scores (see Table 2B). These included the medulla oblongata (11 genes, score = 2.1), thalamus (10 genes, score = 2.0), hypothalamus (nine genes, score = 2.0), hippocampus (nine genes, score = 1.9) and cerebellum (eight genes, score = 1.9). Six (*BDNF*, *DRD2*, C*HRNA7*, *HTR2A*, *SLC6A3*, and *TPH2*) of the overlapping genes were matched to all five tissues types. Sixteen of the overlapping genes matched to a total of 36 Biological Superpathways with a total of nine Superpathways achieving significantly high match scores according to the GeneAnalytics pathway analysis and algorithm [20]. The Circadian entrainment pathway showed the highest match score involving 10 genes (score = 37.0), followed by Amphetamine addiction involving five genes (score = 24.3) and Sudden infant death syndrome (SIDS) susceptibility pathways (six genes, score = 24.1) (see Table 3).

The remaining Superpathways identified did emphasize monoamine signaling and cellular re-uptake/transport. There was little intrinsic overlap between the 36 biological Superpathways with only one gene (*DRD2*) common to all nine Superpathways.

Examination of gene ontology pathways identified 17 genes that matched to a total of 32 GO-molecular functions but high match scores were found for only three molecular functions involving six genes: Serotonin binding with two genes (*HTR2*, *MAOA*) out of nine total pathway genes, score = 14.3; Dopamine binding with two genes (*SLC6A3*, *DRD2*) out of 10 total pathway genes, score = 14) and High Voltage-gated Calcium Channel Activity with two genes (*CACNA1C*, *CACNB2*) out of 10 total pathway genes, score = 14. The 23 overlapping genes mapped to a total of 55 GO-biological processes with high match scores identified for 16 biological processes involving 19 genes (see Table 4). These processes involved a variety of behavioral constructs including axon guidance, synaptic transmission, and particularly the activity of ion channels and dopamine homeostasis.

A total of 106 phenotypes were mapped to the 23 overlapping genes with 36 phenotypes involving 18 genes having high match scores (see Table 5). The highest-matched phenotypes were behavioral despair (four genes, score = 24.8), hypoactivity (seven genes, score = 24.4), abnormal serotonin levels (four genes, score = 24.1) and abnormal response to novel objects (four genes, score = 22.2). Additionally, phenotypes impacted GABAergic neuron morphology, synaptic transmission and response to hypoxia and risk of death.

## 3. Discussion

The molecular and genetic architecture of ASD, BPD and SCH with the identified 23 overlapping candidate susceptibility genes common to the three neuropsychiatric illnesses were analyzed to assess shared etiological factors and phenotypes to facilitate mechanistic understanding and potential development of new treatment approaches. Interestingly, the genetic architecture of the overlapping genes for the three disorders converged on brain structures (e.g., medulla oblongata, thalamus), neurotransmitter systems (e.g., dopamine, serotonin) and signal transduction pathways primarily involved in the regulation of circadian oscillations and sleep disturbances. GO-molecular processes were mapped to the neurotransmitter pathways (dopamine, andserotonin) and ion channels (high voltage–gated calcium channels) implicated in mood, addiction and psychotic disorders with regulatory function and expression in brain centers controlling Circadian entrainment.

**Figure 1:**
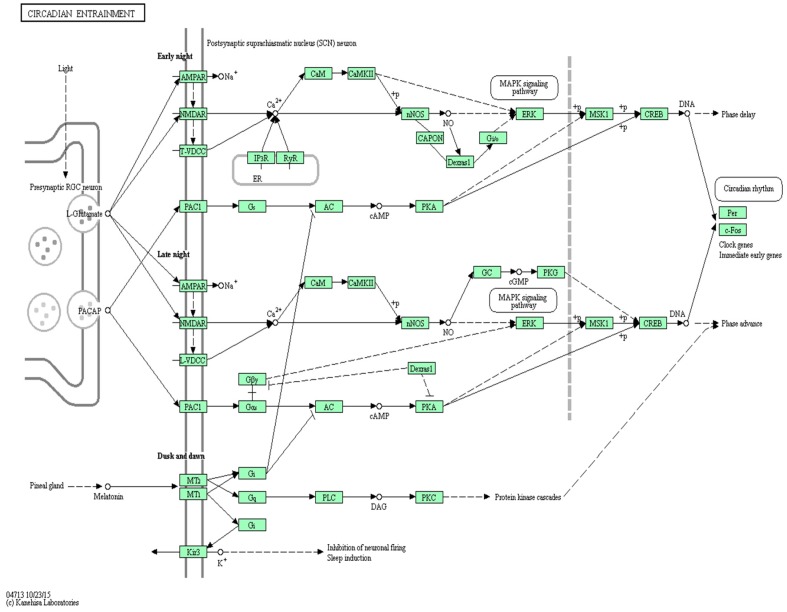
Circadian entrainment is an intrinsic, internal biological clock entrained by exogenous signals, such as endocrine and behavioral rhythms synchronized to environmental cues. The master clock located in the suprachiasmatic nuclei (SCN) of the hypothalamus synchronizes circadian oscillators in peripheral tissues. The main photic input to the suprachiasmatic nuclei comes from the retinal ganglion cells which use glutamate and PACAP, which leads to activation of AMPA and NMDA receptors. The release of glutamate and PACAP triggers the activation of signal transduction cascades including CamKII and nNOS activity, cAMP- and cGMP-dependent protein kinases and mitogen-activated protein kinase (MAPK). Also, melatonin affects non-photic entrainment by inhibiting light-induced phase shifts through inhibition of adenylate cyclase (AC). Additionally, multiple entrainment pathways converge to phosphorylate CREB and to activate *CLOCK* gene expression [28,29,30,31]. Solid and dotted lines indicate direct and indirect relationships, respectively.

The human thalamus, hypothalamus and hippocampus have been extensively targeted in relation to relaying and processing sensory and motor information, as well as regulating consciousness and sleep [24]. The central and peripheral circadian molecular clock is entrained through a complex and highly regulated molecular cascade in the suprachiasmatic nuclei (SCN) of the hypothalamus driven by the cyclical expression of *PER* and *CRY* [25,26,27]. The master lists of susceptibility genes for BPD and SCH contain *CLOCK*, an integral part of the Circadian entrainment pathway, and the identified ASD master gene list contains additional circadian-regulatory genes (e.g., *PER1*, *PER2*, *NPAS2*, *MTNR1A*, and *MTNR1B*). The master clock located in the suprachiasmatic nuclei of the hypothalamus synchronizes mainly by light signals, and releases glutamate and pituitary adenylate cyclase-activating polypeptide (PACAP) with the activation of signal transduction cascades, including nNOS activity, cAMP- and cGMP-dependent protein kinases. Additionally, multiple entrainment pathways converge to phosphorylate CREB and to activate *CLOCK* gene expression [25,26,27] (see Figure 1 for circadian pathways and related features).

Arousal and cortical responsiveness are modulated by a complex regulatory network including serotonergic feedback from the median raphe nuclei involved in the regulation of rapid eye movement (REM) sleep patterning [24]. Depletion of serotonin receptors and feedback are implicated in the pathology of Sudden infant death syndrome (SIDS), a secondary Superpathway identified in our study [32]. *BDNF*, a neurotrophic factor, and *CHRNA7* genes involved in the SIDS Superpathway were also implicated in the biological processes impacting the response to hypoxia and may reflect underlying vulnerability to neurological/physiological injury secondary to apnea, thereby influencing neurocognition and/or behavior. Mesencephalic dopaminergic neurons from the ventral tegmental area also project to the thalamic nuclei to directly modulate sleep induction and wakefulness through opposing effects of *DRD1* and *DRD2* receptors on adenylyl cyclase activity.

Neuronal activity and plasticity during brain development is sensitive to both neurotransmitter signaling of serotonin and dopamine and responsive to common genetic and environmental factors influencing brain maturation. Monoamine-sensitive periods also modulate select neurodevelopmental processes (e.g., neuron division, migration and dendritic connectivity) involved in the development of behavioral regulation and control as well as sleep patterns, which may reflect core etiological relationships linking neuropsychiatric illnesses to sleep disruption and hypoxia [28,33,34,35,36,37,38]. Additionally, the homeostatic value of sleep and circadian rhythmicity impact physiology and behavior in important ways, including the response to stress. Rhythmic circadian oscillations in glucocorticoid levels can modulate adult hippocampal growth and functioning through inhibitory effects of glucocorticoid hormones on neural stem cell and progenitor cell proliferation [39]. Thus, the disruption of circadian entrainment or loss of circadian regulation of glucocorticoid release could directly influence hippocampal neuroplasticity, learning and memory throughout life, which may play a role in psychopathology such as schizophrenia [40,41].

Our analyses are limited by the current status of research and availability of published literature reports on candidate genes as well as the reliability of the curated databases and integrated pathway analyses produced by the GeneAnalytics algorithms. Advances in genomic technology and bioinformatics will continue to identify and characterization new candidate genes, but not all identified genes will be equally important or certain to be causative. The relative contributions of any individual gene to the general disease prevalence must be assessed individually. Further, intrinsic bias in the curated literature may result from imbalances in the allocation of resources for study which may overemphasize some disease states or scientific disciplines over others (e.g., genetics of cancer over psychiatry). Nevertheless, the convergence of these model systems and overlaid genetic mechanisms provides relevant insight into the key macro systems involved in pathogenesis and the overlap of these three severe neuropsychiatric disorders.

## 4. Materials and Methods

We used recently published list of genes found to be clinically relevant and known to play a role in ASD [21], bipolar disorder [22] and schizophrenia [23] for molecular profiling and pathway analysis of genes common to all three neuropsychiatric conditions with similar features. GeneAnalytics (http://geneanalytics. genecards.org/ [20]) computer program and genomic databases are part of the GeneCards Suite developed by LifeMap Sciences (http://www.lifemapsc.com/products/genecards-suite-premium-tools/) and were used to map the resultant list of common genes to characterize molecular pathways, biological processes, molecular functions, phenotypes, tissues and cells, diseases and compounds affected by overlapping neuropsychiatric genes.

GeneAnalytics is powered by GeneCards, LifeMap Discovery, MalaCards and PathCards, which combine >100 archived data sources [20]. The databases contain gene lists for tissues and cells, diseases, phenotypes pathways and compounds curated from published literature reports to develop the best matched list of genes, scored and subdivided into their biological categories such as diseases or pathways. These applications are integrated with GeneCards human gene database, Malacards human disease database, PathCards, human biological pathways database, and LifeMap Discovery tissues and cells database in order to provide an extensive universe of data from human genes, proteins, cells, biological pathways, diseases and their relationships with integration valuable for research and discovery purposes.

Disease matching scores were derived based upon the number of overlapping genes found and the nature of the gene-disease associations. Tissues and cells were scored using a matching algorithm that weighs tissue specificity, abundance and function of the gene. Related pathways were then grouped into Superpathways to improve inferences and pathway enrichment, reduce redundancy and rank genes within a biological mechanism via the multiplicity of constituent pathways with the methodology and algorithm generated by the GeneAnalytics computer-based program. Superpathways were scored based upon transformation of the binomial *p*-value which was equivalent to a corrected *p*-value with significance defined at <0.0001.

## 5. Conclusions

Genetic overlap among autism spectrum disorder, bipolar disorder and schizophrenia was most strongly mapped to Superpathways and brain tissue types guiding Circadian entrainment. The results illustrate the converging effects of dopamine, serotonin and the signal transduction pathways involved in mood, behavior, cognition and impaired social functions; learning and memory are affected in these neuropsychiatric disorders which also guide brain development and sleep patterning disturbances. Thus, under-recognized sleep dysregulation as a common component of psychiatric illness appears to reflect the underlying molecular and genetic architecture of disease pathology in psychiatric illnesses. The convergence of pathways governing circadian rhythms supports the existence of a common core etiological relationship between neuropsychiatric illness and sleep disruption possibly related to central brain stem dysfunction impacting the presentation and underlying pathology and course of illness. This observation opens a new avenue to pursue for treatment modalities in order to change the clinical outcome and natural history of those affected with these relatively common mental health disorders in our current society.

## Figures and Tables

**Table 1:** Twenty-three clinically relevant genes common to ASD, BPD and schizophrenia.

Gene Symbol	Gene Name	Chromosome Location
*BDNF*	Brain-derived neurotrophic factor	11p14.1
*ANK3*	Ankyrin 3	10q21.2
*CACNA1C*	Ca^2^+ channel, voltage-dependent, L type, α1C subunit	12p13.33
*CACNB2*	Ca+ channel, voltage dependent, β2 subunit	10p12.33
*CHRNA7*	Cholinergic receptor, nicotinic, α7 (neuronal)	15q13.3
*CNTNAP5*	Contactin associated protein-like 5	2q14.3
*CSMD1*	CUB and sushi multiple domains 1	8p23.2
*DISC1*	Disruption in schizophrenia 1	1q42.2
*DPP10*	Dipeptidyl-peptidase 10 (non-functional)	2q14.1
*DRD2*	Dopamine receptor D2	11q23.2
*FOXP2*	Forkhead box P2	7q31.1
*GSK3B*	Glycogen synthase kinase 3β	3q13.33
*HTR2A*	5-Hydroxytryptamine (serotonin) receptor 2A, G-protein-coupled	13q14.2
*MAOA*	Monoamine oxidase A	Xp11.3
*MTHFR*	Methylenetetrahydrofolate reductase	1p36.22
*NOS1AP*	Nitric oxide synthase 1 (neuronal) adaptor protein	1q23.3
*NRG1*	Neuregulin 1	8p12
*PDE4B*	Phosphodiesterase 4B, CAMP-specific	1p31.3
*SLC6A3*	Solute carrier family 6 (neurotransmitter transporter, dopamine), member 3	5p15.33
*SYN3*	Synapsin III	22q12.3
*TCF4*	Transcription factor 4	18q21.1
*TPH2*	Tryptophan hydroxylase 2	12q21.1
*ZNF804A*	Zinc finger protein 804 A	2q32.1

**Table 2:** GeneAnalytics program mapping of diseases, tissues and cells that were significantly matched to 23 overlapping genes for autism spectrum disorder, bipolar disorder and schizophrenia.

**A. Diseases**	**Genes Matched to Disease Type (Highmatch Score)**	**No. of Genes in Disease Type**	**Score**
Schizophrenia	*ANK3*, *BDNF*, *CACNA1C*, *CHRNA7*, *DISC1*, *NRG1*, *DRD2*, *GSK3B*, *HTR2A*, *MAOA*, *MTHFR*, *NOS1AP*, *PDE4B*, *SLC6A3*, *SYN3*, *TPH2*, *ZNF804A*	249	15.1
	(Mediummatch scores > 6.0)		
Bipolar disorder	*BDNF*, *DISC1*, *DRD2*, *GSK3B*, *HTR2A*, *MAOA*, *NRG1*, *SLC6A3*, *ZNF804A*	39	9.6
Autism spectrum disorder	*BDNF*, *CHRNA7*, *DISC1*, *DRD2*, *FOXP2*, *HTR2A*, *MAOA*, *MTHFR*, *SLC6A3*, *TPH2*	103	9.1
Disease of mental health	*BDNF*, *DISC1*, *DRD2*, *HTR2A*, *MAOA*, *NRG1*, *SLC6A3*, *ZNF804A*	57	8.0
Attention deficit hyperactivity disorder	*BDNF*, *CHRNA7*, *DRD2*, *HTR2A*, *MAOA*, *SLC6A3*, *TPH2*, *ZNF804A*	63	7.8
Mood disorder	*BDNF*, *CACNA1C*, *DISC1*, *DRD2*, *HTR2A*, *MAOA*, *TPH2*	31	7.7
Psychotic disorder	*BDNF*, *CHRNA7*, *DISC1*, *DRD2*, *HTR2A*, *NRG1*, *SLC6A3*	37	7.5
Anxiety disorder	*BDNF*, *DRD2*, *HTR2A*, *MAOA*, *SLC6A3*, *TPH2*	21	7.1
Obsessive compulsive disorder	*BDNF*, *DRD2*, *HTR2A*, *MAOA*, *SLC6A3*, *TPH2*	29	6.8
Personality disorder	*DRD2*, *HTR2A*, *MAOA*, *SLC6A3*, *TPH2*	14	6.4
**B. Tissues and Cells**	**Genes Matched to Tissues and Cells**	**No. of Genes in Tissues And Cells**	**Score**
Medulla oblongata	*BDNF*, *CHRNA7*, *DRD2*, *FOXP2*, *HTR2A*, *MAOA*, *NRG1*, *PDE4B*, *SLC6A3*, *TCF4*, *TPH2*	2179	2.1
Thalamus	*BDNF*, *CHRNA7*, *DRD2*, *HTR2A*, *MTHFR*, *PDE4B*, *SLC6A3*, *TCF4*, *TPH2*	1736	2.0
Hypothalamus	*BDNF*, *CHRNA7*, *DRD2*, *FOXP2*, *HTR2A*, *MTHFR*, *PDE4B*, *SLC6A3*, *TCF4*, *TPH2*	1666	2.0
Hippocampus	*ANK3*, *BDNF*, *CHRNA7*, *DISC1*, *DRD2*, *FOXP2*, *HTR2A*, *SLC6A3*, *TPH2*	3335	1.9
Cerebellum	*ANK3*, *BDNF*, *CHRNA7*, *DRD2*, *FOXP2*, *HTR2A*, *SLC6A3*, *TPH2*	2609	1.9

**Table 3:** GeneAnalytics program mapping of superpathways with high match scores for 23 overlapping genes for autism spectrum disorder, bipolar disorder and schizophrenia.

Superpathways	Genes Matched to Superpathways	No. of Genes in Superpathways	Score
Circadian entrainment	*SLC6A3*, *GSK3B*, *HTR2A*, *MAOA*, *NOS1AP*, *PDE4B*, *TPH2*, *CACNA1C*, *CHRNA7*, *DRD2*	390	37.0
Amphetamine addiction	*SLC6A3*, *MAOA*, *BDNF*, *CACNA1C*, *DRD2*	87	24.3
SID susceptibility pathways	*HTR2A*, *MAOA*, *NOS1AP*, *TPH2*, *BDNF*, *CHRNA7*	185	24.1
Selective serotonin reuptake inhibitor pathways	*HTR2A*, *MAOA*, *TPH2*	29	17.5
Monoamine transport	*SLC6A3*, *MAOA*, *TPH2*	36	16.6
Transmission across chemical synapses	*SLC6A3*, *MAOA*, *SYN3*, *CACNB2*, *CHRNA7*	316	15.2
CREB pathways	*HTR2A*, *NRG1*, *BDNF*, *CACNA1C*, *CACNB2*, *CHRNA7*	562	14.9
Neurotransmitter clearance in the synaptic cleft	*SLC6A3*, *MAOA*	8	14.6
CAMP signaling pathways	*PDE4B*, *BDNF*, *CACNA1C*, *DRD2*	211	13.4

**Table 4:** GeneAnalytics program mapping of gene ontology (GO) biological processes with high match scores to 23 overlapping genes for autism spectrum disorder, bipolar disorder and schizophrenia.

GO-Biological Processes	Genes Matched to GO-Biological Processes	No. of Genes in GO-Biological Processes	Score
Startle response	*NRG1*, *CSMD1*, *DRD2*	20	19.1
Positive regulation of axon extension	*GSK3B*, *NRG1*, *DISC1*	30	17.4
Cellular calcium ion homeostasis	*HTR2A*, *CACNA1C*, *CHRNA7*, *DRD2*	107	17.2
Synaptic transmission	*SLC6A3*, *HTR2A*, *MAOA*, *CACNA1C*, *CACNB2*, *CHRNA7*	432	17.0
Dopamine catabolic process	*SLC6A3*, *MAOA*	5	16.0
Axon guidance	*GSK3B*, *NRG1*, *ANK3*, *BDNF*, *CACNA1C*, *CACNB2*	537	15.3
Synapse assembly	*NRG1*, *BDNF*, *DRD2*	52	15.0
Regulation of high voltage-gated calcium channel activity	*NOS1AP*, *PDE4B*	7	15.0
Regulation of potassium Ion transport	*ANK3*, *DRD2*	7	15.0
Response to hypoxia	*MTHFR*, *BDNF*, *CHRNA7*, *DRD2*	180	14.3
Negative regulation of synaptic transmission, glutamatergic	*HTR2A*, *DRD2*	9	14.3
Adenohypophysis development	*SLC6A3*, *DRD2*	9	14.3
Regulation of synaptic transmission, GABAergic	*SYN3*, *DRD2*	10	14.0
Behavioral response to ethanol	*CHRNA7*, *DRD2*	11	13.7
Regulation of dopamine secretion	*SLC6A3*, *CHRNA7*	11	13.7
Dopamine biosynthetic process	*HTR2A*, *DRD2*	12	13.4

**Table 5:** GeneAnalytics profiling of high match score phenotypes to 23 overlapping genes for autism spectrum disorder, bipolar disorder and schizophrenia.

Phenotypes	Genes Matched to Phenotypes	No. of Genes	Score
Behavioral despair	*GSK3B*, *CACNA1C*, *CSMD1*, *DISC1*	28	24.8
Hypoactivity	*MAOA*, *FOXP2*, *ANK3*, *BDNF*, *CACNA1C*, *CHRNA7*, *DRD2*	314	24.4
Abnormal serotonin level	*MAOA*, *FOXP2*, *TPH2*, *BDNF*	32	24.1
Abnormal response to novel object	*SLC6A3*, *FOXP2*, *TPH2*, *DISC1*	44	22.2
Abnormal GABAergic neuron morphology	*BDNF*, *CHRNA7*, *DRD2*	11	21.7
Abnormal prepulse inhibition	*NRG1*, *DISC1*, *DRD2*	14	20.7
Abnormal social Investigation	*MAOA*, *SYN3*, *CACNA1C*, *DISC1*	14	20.7
Increase aggression towards males	*MAOA*, *TPH2*, *BDNF*	64	20.1
Small cerebellum	*MTHFR*, *FOXP2*, *ANK3*, *DISC1*	67	19.8
Decrease exploration in new environment	*GSK3B*, *FOXP2*, *BDNF*, *CACNA1C*	79	18.9
Abnormal CNS synaptic transmission	*SLC6A3*, *BDNF*, *CACNA1C*, *DRD2*	79	18.9
Premature death	*SLC6A3*, *MTHFR*, *FOXP2*, *ANK3*, *TPH2*, *BDNF*, *CACNA1C*, *DRD2*	830	18.6
Decrease startle reflex	*FOXP2*, *CSMD1*, *DISC1*, *DRD2*	84	18.6
Decrease anxiety-related response	*HTR2A*, *CHRNA7*, *DISC1*, *DRD2*	89	18.2
Increase dopamine level	*SLC6A3*, *MAOA*, *FOXP2*	31	17.2
Abnormal vocalization	*FOXP2*, *CACNA1C*, *DRD2*	37	16.5
Hyperactivity	*SLC6A3*, *NRG1*, *BDNF*, *CSMD1*, *DISC1*	272	16.3
Increased thigmotaxis	*SLC6A3*, *CACNA1C*, *CSMD1*	45	15.6
Abnormal serotonergic neuron morphology	*TPH2*, *BDNF*	6	15.4
Abnormal response to novel odor	*SLC6A3*, *DRD2*	6	15.4
Abnormal latent inhibition of conditioning	*DISC1*, *DRD2*	6	15.4
Impaired coordination	*SLC6A3*, *FOXP2*, *BDNF*, *CACNA1C*, *DRD2*	309	15.4
Abnormal learning/memory/conditioning	*GSK3B*, *CACNA1C*, *DISC1*	49	15.3
Limp posture	*NRG1*, *DRD2*	7	15.0
Decreased serotonin Level	*TPH2*, *DISC1*	7	15.0
Postnatal growth retardation	*SLC6A3*, *MTHFR*, *FOXP2*, *TPH2*, *BDNF*, *DRD2*	581	14.6
Abnormal pituitary gland physiology	*SLC6A3*, *DRD2*	8	14.6
Abnormal inhibitory postsynaptic currents	*SLC6A3*, *SYN3*, *BDNF*	60	14.4
Small nodose ganglion	*NRG1*, *BDNF*	9	14.3
Complete postnatal lethality	*FOXP2*, *ANK3*, *TCF4*, *BDNF*, *CACNA1C*	375	14.1
Small petrosal ganglion	*NRG1*, *BDNF*	10	14.0
Decreased somatotroph cell number	*SLC6A3*, *DRD2*	11	13.7
Abnormal grooming behavior	*SLC6A3*, *MAOA*	11	13.7
Abnormal excitatory postsynaptic currents	*NRG1*, *SYN3*, *DRD2*	72	13.6
Decreased left ventricle systolic pressure	*MAOA*, *NRG1*	12	13.4
Hunched posture	*MAOA*, *BDNF*, *DRD2*	78	13.3

## Abbreviations

ASD	Autism spectrum disorder
BPD	Bipolar disorder
SCH	Schizophrenia
GWAS	Genome-wide association studies
CGH	Comparative genomic hybridization
CNV	Copy number variants
SIDS	Sudden infant death syndrome
SCN	Suprachiasmatic nuclei
PACAP	Pituitary adenylate cyclase-activating polypeptide
cAMP	Cyclic adenosine monophosphate
cGAMP	Cyclic guanosine monophosphate–adenosine monophosphate
nNOS	Nitric oxide synthase
CREB	cAMP response element-binding protein
